# Firearm Safety in a Country of Arms

**DOI:** 10.1111/1468-0009.70091

**Published:** 2026-05-21

**Authors:** JONATHAN M. METZL

**Affiliations:** ^1^ Vanderbilt University

## Abstract

**Policy Points**

- Firearm safety policy in the United States cannot succeed through legislation alone; effective interventions must also address the social, economic, and infrastructural conditions that shape perceptions of safety.
- Evidence suggests that place‐based investments can reduce violence and firearm deaths while strengthening social cohesion and civic trust.
- Public health approaches to firearms often fail to resonate across political divides because many Americans view guns not simply as health risks, but as symbols of personal liberty and self‐protection.
- Durable firearm safety policy will likely require bipartisan strategies that combine targeted gun‐safety measures with broader investments in community safety, economic stability, and public infrastructure that people experience in everyday life.

In april 2018, a naked man with an ar‐15 burst into a waffle house restaurant in Nashville, Tennessee.^1^ Firing at random, he murdered four people and gravely injured five more before escaping into the night.

The 27‐year‐old shooter was captured several days later, and it was quickly revealed that he displayed a years‐long history of unstable behavior. His violent and potentially psychotic tendencies had grown abundantly clear to family members and police. Yet he was still able to buy and carry semiautomatic weapons, including the one he used in the mass shooting.

Surely it was time for Tennessee to pass firearm safety laws. Or so gun safety supporters contended in the aftermath of the tragedy. Democratic politicians, gun‐safety experts and activists, and survivors and family members of the victims took to the airwaves and to the floor of the Tennessee statehouse demanding change. Their claims were bolstered by research showing that effective gun laws save lives.^2^ Harm reduction strategies like Extreme Risk Protection Orders (ERPOs), safe storage guidelines, or background checks conceivably could have stopped this shooting and so many others.^3^, ^4^ Like most US red states, Tennessee had few such laws on the books.

Instead, the opposite scenario played out. No gun‐safety legislation passed into law. In a gubernatorial election held months after the tragedy, voters overwhelmingly elected the most pro‐gun governor in state history, who ran on a platform that championed eliminating the few remaining state restrictions on gun ownership as well as permits and regulations policing firearms in public places.^5^, ^6^ It became even easier to buy and carry firearms as a result.^7^

Why do growing numbers of American states fail to enact comprehensive gun safety interventions, even when the public health implications of not doing so appear clear? Strengthening gun laws in the aftermath of shootings often seems a self‐evident response across much of the world.^8^, ^9^ Data repeatedly show that when countries like Britain, Australia, Canada, New Zealand, and Norway tighten gun laws and promote gun‐safety policies, it leads to less gun violence and fewer mass shootings.^10^, ^11^, ^12^, ^13^

In the United States, however, mass shootings produce divergent and often polarizing responses.^14^ Gun‐safety advocates—often Democrats, liberals, academics, and grassroots organizations—promote laws and policies.^15^ Gun‐rights advocates and organizations—often Republicans, conservatives, lobbying organizations like the National Rifle Association (NRA), and gun‐rights groups—respond by pushing for expanding access to guns and eliminating regulations.^16^

This partisan divide presents a particular challenge from a policy perspective. Over the past decade, academic literature has convincingly shown that, when implemented, public health–based approaches can reduce firearm‐related injuries and deaths, promote community safety, and lower health care costs. Lee and colleagues established that stronger overall gun policy environments, and especially permit‐to‐purchase and strengthened background checks, are associated with fewer shootings.^17^ Siegel and colleagues found that universal background checks and prohibitions for violent misdemeanors are associated with an ≈15%–18% reduction in homicides, while permissive “shall‐issue” concealed‐carry laws lead to an ≈9% increase in homicides.^18^ A cross‐sectional study by Cornell and colleagues correlated stronger gun laws with decreased overall firearm mortality, with the strongest correlations for decreased suicides—lending particular support to policies that regulate firearm sales, transfers, and permitting laws.^19^

Indeed, following the election of a self‐described “gun sense” majority in 2022, Michigan passed significant gun safety reforms including the implementation of ERPO laws, universal background checks, and safe storage requirements. Although the laws are relatively new, advocates point to their potential for long‐term reductions in firearm‐related suicides and homicides.^20^, ^21^

Yet policy implementation largely goes in reverse across much of the United States. Public carry has become law in a growing number of states.^22^ If they make it that far, gun‐safety laws and policies die in committee, while gun rights expand nationwide. Calls for policy reform often go nowhere even after school shootings.^23^ In the aftermath of the US Supreme Court's *Bruen* decision, lower courts have overturned firearm safety laws put in place by voters.^24^, ^25^, ^26^ The Supreme Court of the United States has agreed to review a case that challenges whether states can ban people from carrying guns at privately owned public locales including beaches, bars, and restaurants that serve alcohol.^27^ The US Justice Department meanwhile is weighing a series a series of actions that are expected to ease restrictions on the private sale of guns and loosen regulations on shipping firearms by mail.^28^

The United States is a nation with more guns than people and more shootings than calendar days.^29^, ^30^ According to opinion polls, although the public remains divided, decreasing percentages of people support stricter laws, and growing numbers endorse broadened gun rights.^31^ Support for a ban on handguns has reached near‐record lows.^32^ Gun ownership is rising even among groups that historically support tight gun laws, including self‐described liberals and communities of color.^33^, ^34^, ^35^ “The Hard‐Left Shooters Leading a Gun Culture Revolution” reads a November 2025 Wired headline, above an article about growing communities of “gun‐loving” trans and queer people “in Trump's America.”^36^ Growing numbers of Americans under the age of 18 have endorsed the notion that they are “safer with guns than without them.”^37^

## The Sociology of American Firearms

An emerging body of literature from the social sciences explores how consensus around gun policy is often difficult, not just because of polarized political systems but also because when Americans across the political spectrum talk about gun safety, they are talking about different things.^38^, ^39^, ^40^, ^41^, ^42^ Our inability to find common ground about gun‐related injuries and deaths reveals greater disagreements surrounding safety and civic life. Key themes from that literature include

- divergent frameworks,
- differing notions of threat and safety, and
- differing forms of political engagement.

### Divergent Frameworks

For many academics and liberal Americans, gun‐related injuries and deaths represent a public health crisis.^43^ They describe gun violence as an “epidemic”—a Hippocratic term taken from the study of infectious diseases—best addressed through harm reduction strategies that balance gun rights with the types of regulations used to counter other man‐made threats to mortality such as cigarettes, faulty seatbelts, or asbestos insulation.^44^, ^45^

Many conservative gun owners don't describe it that way. From their perspective, unlike cigarettes or cars, firearms represent enshrined individual rights or liberties that provide safety against criminals, liberals, or the very types of government‐based regulatory laws and policies at the core of public health.^46^, ^47^ The public health framework strikes many conservative gun owners as counterproductive because it contrasts with beliefs about freedom and autonomy.^48^ Among Republicans, support for public health authority broadly, and regarding information about gun safety specifically, began to steadily decline during the COVID‐19 pandemic and has yet to rebound.^49^, ^50^

I've observed such trends in my own research. A man who lived on a farm in Tennessee and owned multiple AR‐15s told me how the idea of the government regulating his liberties—through background checks requiring that he enter personal identification into government databases or civil restraining orders that expanded police authority to take away guns—filled him with anger.^51^, ^52^ “I'm not going along with gun control because the very act of banning or confiscating guns reinforces the reason I own them in the first place,” he said. “And in any case, the government isn't going to save you from violence. Each individual is their own most effective first responder.”

Research suggests that liberal gun owners have their own complex reasons for buying and carrying firearms. Drawing on General Social Survey data, Yamane and colleagues found that liberal gun owners constitute a genuinely distinct group, differing from conservative gun owners in important ways, including religious affiliation, religiosity, and punitive attitudes.^53^ Hubbert and Eaton revealed that liberal gun owners tend to frame ownership in collective rather than individualist terms, emphasizing democratic accountability and community belonging rather than personal sovereignty.^54^ A Boston University study by Boine and colleagues further complicates the picture by identifying a “Gun Culture 3.0”—an emphasis on Second Amendment activism—that has grown most rapidly in states with stricter gun laws, suggesting that the political meanings of gun ownership are shifting in ways that cut across traditional partisan lines.^55^

All of this points toward a core challenge facing public health: a gap between what guns do and what they mean. Public health focuses on reducing the nearly 50,000 American gun deaths per year. But methods meant to prevent injuries and deaths have relatively less to say about the roughly 500 million guns owned and carried by people across the United States, or the vast majority of gun owners who are never involved in shootings or crimes.^56^, ^57^, ^58^

As a disease‐based model, public health often implicitly links guns with unhealthy outcomes in ways that render it a less credible frame when talking about gun ownership more broadly. In my research, I detail how the gap between framing guns as health risks and as viable tools of public life is then filled by self‐interested parties like gun manufacturers, who cast gun ownership as a trapping of identity through themes of protection and liberty, while at the same time playing on fears about violence and unrest. Guns increasingly become responses to feelings of uncertainty in ways that go hand in hand with rising gun sales and falling support for authorities, community safety efforts, or democratic institutions.^59^, ^60^, ^61^, ^62^, ^63^, ^64^

On a practical level, vast increases in the number of public guns render it nearly impossible for safety experts to pick out the potential shooters of the world from the millions of people who own and carry firearms for personal protection, hunting, family tradition, or a host of other reasons, and whose guns are never fired in shootings or crimes.^65^ And at the level of ideology, selling and promoting guns during moments of peril bolsters a neoliberal belief long undergirding gun rights arguments—that your safety is your responsibility because the state won't protect you. This belief renders a central tenet of the public health approach increasingly illogical: the notion that equitable regulations and collective policies yield trust and well‐being among people.^66^

### Differing Notions of Threat and Safety

Liberal networks often call for “common sense” gun reform after mass shootings.^67^, ^68^ The language evokes profound emotional resonance; gun death and suffering feel nonsensical and harrowing. Mobilizing in the aftermath of these horrific events also channels attention: These are the heightened moments when many non‐gun‐owners think about guns.

Talk to conservative gun owners for more than a minute, however, and it quickly becomes evident that mass shootings occupy relatively little space in their formulations. Rather, they frequently cite concerns about robbery or homicide as among the primary reasons for common sense gun ownership. “The bad guys have guns, so why shouldn't we?” is a common refrain. Regulation codes as risk because it puts law‐abiding “good guys” at a disadvantage against “bad guys” who do not play by the same rules.^69^

Such assumptions are often replete with stereotypes. A red state gun owner explained to me that Southern conservatives remain skeptical of regulation because “the majority of violent gun crime is committed in places with the strictest control on guns”—in other words, in dense, urban blue cities by persons of color.^70^, ^71^ Moreover, these claims often overlook how permissive guns laws themselves are associated with higher rates of certain types of violence,^72^, ^73^ and how conservative gun‐owning populations see disproportionally high rates of firearm suicide.^74^

But they are not wholly wrong. Democrats increasingly moved away from expressly linking gun reform to crime reduction in the decades following the notorious 1994 crime bill, which led to profoundly disparate racial outcomes.^75^, ^76^ Public health now frequently casts murders, homicides, and robberies as the responsibility of police, not of gun safety reform. Whether methods designed to treat guns as public health risks directly lower crime rates is debatable.^77^

This divergence often casts Republicans and conservatives as credible messengers on rule of law, while putting Democrats, liberals, and progressives at electoral risk.^78^, ^79^ Justified or overblown, growing numbers of people even in blue cities and states cite concerns about lawlessness and crime.^80^ Then‐Republican Party candidate Lee Zeldin fanned fears about subway crime and advocated for expanded gun rights in a surprisingly competitive race for the New York governor's office in 2022. Former sheriff Dave Reichert did the same in 2024 as a gubernatorial candidate in historically blue Washington state.^81^ Donald Trump calls blue cities “hell holes” in support of efforts to roll back gun safety laws across the United States.^82^, ^83^

Complicating matters further, gun laws themselves can convey punitive implications and effects. Writing about well‐intentioned laws that make improper firearm storage a felony, public health scholar Caitlin McMurtry argues that “public health messages and policies that fail to respond to the root motivations for armament and that attempt to encourage behavior change by threatening incarceration may heighten distrust among gun owners and create incentives to hide one's behaviors from providers and national surveys” (p. 984).^84^

Policy scholars need not go down every fraught rabbit hole. Yet reluctance to address crime exposes potential gaps in public health formulations that might be more directly addressed by studying whether specific gun safety policies lower specific types of crime,^85^ by researching and promoting effective models of policing,^86^ or by vocally countering Republican policies that clearly do increase crime, such as the disastrous “guns in trunks” legislation in Tennessee that has led to skyrocketing car break‐ins and gun thefts.^87^

### Differing Forms of Political Engagement

Over the past decades, Democratic formulations effectively shifted mainstream opinion toward gun safety. Harm‐reduction strategies such as background checks and assault rifle bans grew increasingly popular with majorities of Americans.^88^ ^,^ ^89^

However, supporting policies in opinion polls is different from voting for them. Data suggest that interest in gun reform peaks in the four days following mass shootings but then wanes, and often fails by itself to yield better voter turnout.^90^ By contrast, conservative gun owners vote reliably for pro‐gun Republican candidates and punish politicians who make even modest moves toward reform.^91^, ^92^

Across many red and purple states, years of mobilizing voter blocs around gun rights have allowed the Republican Party to control key legislative bodies, appoint and elect loyal politicians, seat sympathetic judges, build donor bases, gerrymander districts, and accrue other levers of authority in ways that grow immune to shifts in popular opinion.^93^, ^94^, ^95^, ^96^

Gun safety advocates, emergency room doctors, and pastors representing nearly 800 faith‐based organizations stood on the steps of the state capital in Tennessee in 2021 to protest legislation that would allow state residents to carry handguns without a permit. “The permitless carry of firearms that can take life unjustly is immoral,” one pastor said. An alleged 84% of Tennesseans disproved of the bill. But these protests were unsuccessful, their loss a foregone conclusion. As gun sales surged and shootings rose dramatically in the state, even centrist Republicans felt powerless to change course. “I'm a lifelong Republican and strongly 2A,” a Nashville‐based medic told me at the time, using shorthand for being pro‐Second Amendment. “But this isn't what I signed up for.”^97^, ^98^, ^99^, ^100^

Parents, activists, and politicians again mobilized forcefully in the aftermath of a 2023 mass shooting at the Covenant School in Nashville.^101^ But gerrymandered power and political and social influence—what political scientist Matthew Lacombe calls firepower—made it increasingly difficult for citizens to pass legislation or contest policies with which they disagreed.^102^, ^103^, ^104^

## The Structural Competency of Firearm Safety

We inhabit a moment in which divisions grow alongside declining faith in institutions. Yet emerging research in the United States suggests that potential violence can be countered, not just with effective laws or divisive debates but also through investment in infrastructure and lived environment. If US gun proliferation carries larger implications, so too do its potential solutions.^105^

No doubt, short‐term policy change remains possible. As McMurtry rightly puts it, “attempts to pass narrowly tailored policies favored by gun owners and gun nonowners alike, such as ‘red flag’ laws, safety training requirements, and purchase exclusions for violent offenders may prove successful… Bipartisan solutions will be needed as long as firearm sales and gun deaths remain high in the United States” (p. 986).^84^

But bipartisanship is often elusive in the present moment, and policy alone is not enough. From a structural perspective, gun laws and policies by themselves will have relatively little effect in changing the contours of the American gun debate if they don't accompany material investments that take seriously people's safety concerns and reward community cohesion over armed tribalism.^106^, ^107^, ^108^

In 2017, our research team published a study titled “Structural Competency and the Future of Firearm Research,” which concluded that firearm safety efforts needed to expand to more fully address the upstream social factors and inequities that fuel gun violence.^109^ The research advocated policy and intervention strategies that tackle root causes of violence rather than just symptoms of it, and investment in structures that support safety and community in everyday life. We argued for addressing gun proliferation and firearm‐related violence in the context of societal conditions such as disinvestment in communities and economic precarity.

Our study contributed to a growing body of data showing that structural interventions such as fixing streetlights; creating parks and affordable housing; building functioning healthcare systems, green space, and jobs programs; and rebuilding “civic infrastructure,” public safety, and community resiliency can reduce gun crime at rates that supersede those produced by gun laws alone.^110^, ^111^, ^112^, ^113^, ^114^, ^115^

A 2017 review of community programs across 264 US cities found that for every 10 additional nonprofit crime‐reduction programs per 100,000 residents, homicide rates fell by 9% and overall violence by 6%—evidence that community‐level investments, rather than enforcement‐based approaches, can drive meaningful reductions in gun violence.^116^ Built environment literature reinforces this point: a landmark cluster‐randomized trial found that greening vacant lots reduced overall crime, gun assaults, and burglaries compared to untreated lots, and that residents living near greened lots also reported decreased fear and increased outdoor social activity.^117^, ^118^ And a randomized controlled trial in New York City found that improved street lighting in disadvantaged communities reduced serious crime by roughly 40%, with benefits persisting even after three years.^119^

The National Strategy for Suicide Prevention also explicitly endorses upstream policies—including economic stability, housing security, and employment services—as central components of a comprehensive suicide prevention framework, complementing rather than replacing crisis intervention and treatment efforts.^120^ It does so for good reason: economic recession and unemployment are consistently associated with elevated rates of suicidal behavior at both population and individual levels, and research suggests that unemployment benefits, employment protection legislation, higher minimum wages, and active labor market programs may reduce these risks.^121^, ^122^

Such findings support the argument that place‐based and community investments address the structural conditions that produce gun violence, rather than its symptoms. They also suggest the importance of further shifting health policy toward interventions that people can see and feel in their daily lives. In the United States, many people encounter public health most visibly in moments of crisis, such as after mass shootings or during pandemics.^123^, ^124^ Across red‐state America in particular, public health can in this context can connote restricting freedoms or being forced to comply with mandates but rarely represents prosperity in ways that are evident and tactile in the everyday.^125^, ^126^, ^127^ Meanwhile, mistrust—of governments, mandates, police, criminals, or others—taps into larger social and economic fault lines in ways that render the kinds of community participation vital for many public health interventions difficult.

Investments in what sociologist Patrick Sharkey calls “place” can reduce gun crime and build social cohesion. At the level of policy, this might mean joining gun safety researchers with builders, employers, developers, internet providers, supermarkets, transit experts, and city planners to redefine and construct safe neighborhoods.^128^, ^129^ Or by aligning with local and national businesses to promote ways that gun safety can stimulate economic growth. Or perhaps by working with artificial intelligence developers to reimagine safe parks, workplaces, or schools; or rewarding communities to improve public safety using reinforcement algorithms developed by health insurers.^130^, ^131^

These types of interventions take at face value the core assumption of many gun‐rights arguments—that public spaces should be safe for everyone—and intervene upstream to do so. Few of these interventions involve mandates governing individual‐level behaviors. Instead, they aim to engage broader categories of shareholders to build what political theorists call “strategy influence” by creating long‐term sustainable relationships that provide real‐world benefits.^132^

Ultimately, gun safety efforts need to allow people to imagine broader coalitions based on shared interests rather than focus on tribalizing resentments, anxieties, and fears.

As politicians and policy experts in the United States rightly continue to push for basic safety laws, it's important to keep in mind that guns are oft‐lethal matters unto themselves; they are also symbols, material objects, and enforcers in a larger fight about America, who we are, and what we've become.
